# Enhanced DPPH radical scavenging activity and DNA protection effect of litchi pericarp extract by *Aspergillus awamori* bioconversion

**DOI:** 10.1186/1752-153X-6-108

**Published:** 2012-09-27

**Authors:** Sen Lin, Bao Yang, Feng Chen, Guoxiang Jiang, Qing Li, Xuewu Duan, Yueming Jiang

**Affiliations:** 1 Key Laboratory of Plant Resource Conservation and Sustainable Utilization, South China Botanical Garden, Chinese Academy of Sciences, Guangzhou, 510650, People’s Republic of China; 2 Graduate School of Chinese Academy of Sciences, Beijing, 100039, People’s Republic of China; 3 Department of Food, Nutrition and Packaging Sciences, Clemson University, Clemson, SC, 29634, USA

**Keywords:** Litchi pericarp, Phenolics, *Aspergillus awamori*, DPPH radical scavenging activity, DNA protection, HPLC

## Abstract

**Background:**

Litchi (*Litchi chinensis* Sonn.) pericarp is a major byproduct which contains a significant amount of polyphenol. This study was designed to biotransformation litchi pericarp extract (LPE) by *Aspergillus awamori* to produce more bioactive compounds with stronger antioxidant activities.

**Results:**

The study exhibited that the 2,2-diphenyl-1-picrylhydrazyl radical scavenging activities significantly (p < 0.05) increased from 15.53% to 18.23% in the water-extracted fraction and from 25.41% to 36.82% in the ethyl acetate-extracted fraction. Application of DNA cleavage assay further demonstrated the enhanced protection effect of the fermented phenolics on DNA damage. It is also noted that the water-extracted fraction of the fermented LPE possessed a much stronger capacity than the ethyl acetate-extracted fraction to prevent from damage of supercoiled DNA. Interestingly, it was found that some new compounds such as catechin and quercetin appeared after of *A. awamori* fermentation of LPE, which could account for the enhanced antioxidant activity.

**Conclusion:**

The DPPH radical scavenging activity and DNA protection effect of LPE were increased by *Aspergillus awamori* bioconversion while some compounds responsible for the enhanced antioxidant activity were identified. This study provided an effective way of utilizing fruit pericarp as a readily accessible source of the natural antioxidants in food industry and, thus, extended the application area such as fruit by-products.

## Background

Phenolic compounds as secondary metabolites are widely present in fruits and vegetables. A positive relationship between consumption of food with abundant phenolics and low incidence of degenerative diseases, including cancer, heart disease, inflammation, arthritis, brain dysfunction and cataracts, has been confirmed [1]. Litchi (*Litchi chinensis* Sonn.) is a subtropical fruit with a brightly red pericarp which contains a significant amount of phenolics such as epicatechin, procyanidins, cyanidin-3-glucoside, and quercetin-3-rutinoside [2]. These phenolics exhibit good antioxidant ability [3], and anticancer and immunomodulatory activities [4].

Currently, biotransformation of plant byproducts by microorganisms is one of an effective means to produce bioactive compounds. Many fruit byproducts, such as grape pomace, pineapple waste, citrus peel and banana peel, have been used to produce gluconic acid, carotenoids and citric acid by microorganism biotransformation [5-7]. Botella et al. [8] have used grape pomace as a solid substrate to produce xylanase and pectinase. Furthermore, grape pomace could induce the production of laccase for *Trametes versicolor*[9]. Some species of microorganisms belonging to the *Aspergillus* genus can produce tannase [10], naringinase [11], β-glucosidase [12], feruloyl esterase [13] and flavonol 2,4-dioxygenase [14], which make it possible to transform bioactive compounds. Hung et al. [15] have found that the antimutagenic activity of black bean can be increased after the treatment with *Aspergillus. awamori*. In the present work, *A. awamori* was chosen as a fermented microorganism to biotransformed litchi pericarp extract (LPE). The changes in the content and antioxidant activity of bioactive compounds in litchi pericarp before and after biotransformation by *A. awamori* were investigated.

## Results

### Contents of total phenolics and flavonoids

**Figure 1:**
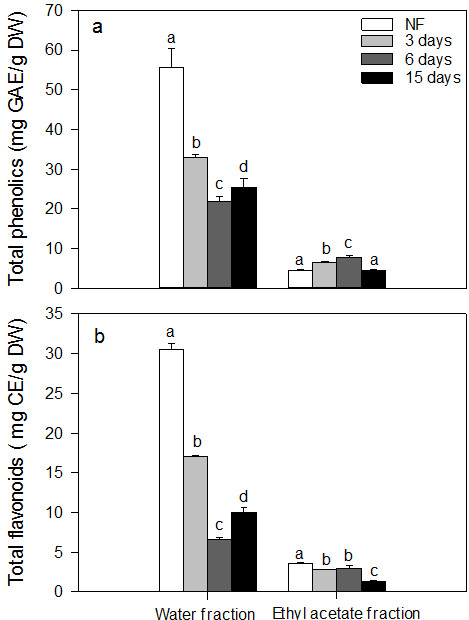
**Contents of total phenolics (a) and total flavonoids (b) in the non-*A. awamori*-fermented LPE (NF) and *A. awamori*-fermented LPE after 3 days, 6 days and 15 days.** Each value represents the means ± SD (n = 3).

As shown in Figure 1, the phenolic content of the water fraction of LPE decreased and then increased on the 15^th^ day in the fermentation period. On the contrary, the phenolic content of the ethyl acetate fraction of LPE decreased, then increased on the 6^th^ day and finally declined on the 15^th^ day. Similarly, a change in the flavonoid content of the water fraction was observed. In addition, when the fermentation with *A. awamori* on the Czapek-Dox medium without LPE was conducted, very small amounts of phenolics (i.e., 0.121, 0.093 and 0.046 mg GAE/ml extract in the water fraction and 0.081, 0.073 and 0.066 mg GAE/ml extract in the ethyl acetate fraction on the 3^rd^, 6^th^ and 15^th^ day, respectively) were observed. However, no flavonoids can be detected in either the water fraction or the ethyl acetate fraction.

### Scavenging activity of DPPH radical

**Figure 2:**
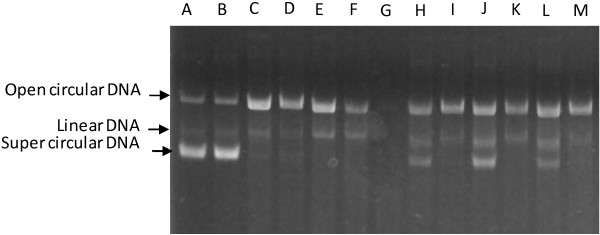
**HPLC chromatograms of the *A. awamori*-fermented (F) or non-*Aspergillus awamori*-fermented (NF) LPE.** Chromatogram (**a**), the water fraction of non-*A. awamori*-fermented LPE; Chromatogram (**b**), the water fraction of *A. awamori*-fermented LPE; and Chromatogram (**c**), the ethyl acetate fraction of the *A. awamori*-fermented LPE.

As shown in Table 1, a great change in the DPPH radical scavenging activity occurred after fermentation with *A. awamori.* Maximum value of DPPH radical scavenging activity of the water fraction of LPE was achieved after 3 days of fermentation, and then declined, which was associated with reduced phenolic content. The DPPH radical scavenging activity of the ethyl acetate fraction of LPE decreased within the first 6 days of fermentation, and then dramatically increased on the 15^th^ day. However, a poor DPPH radical scavenging activity was observed when fermented by *A. awamori* on the Czapek-Dox medium without LPE (data not shown). Moreover, no significant change in the DPPH scavenging activity of litchi pericarp extract in the absence of *A. awamori* was observed (data not shown) in the culture period. Thus, concentration and composition of phenolic compounds greatly affect the DPPH radical scavenging activity of LPE in this study. HPLC analysis showed that catechin (peak 6), a unidentified compound (peak 7) and quercetin (peak 6’) appearing on the 3^rd^, 6^th^ and 15^th^ day during fermentation (Figure 2) may account for the increased DPPH radical scavenging activity of the *A. awamori*-fermented LPE.

**Table 1:** DPPH radical scavenging activity of LPE

	**DPPH radical scavenging activity (%)**	
	**Water fraction**	**Ethyl acetate fraction**
3 days-*A. awamori*-fermented LPE	18.23 ± 1.06^a^	19.5 ± 1.12^a^
6 days-*A. awamori*-fermented LPE	11.86 ± 1.10^b^	15.43 ± 1.45^b^
15 days-*A. awamori*-fermented LPE	12.53 ± 1.16^b^	36.82 ± 2.19^c^

DPPH radical scavenging activities of LPE in the water-extracted fraction and ethyl acetate-extracted fraction in the absence of A. awamori were 15.53 ± 1.36 and 25.41 ± 1.78, respectively. Means followed by the same letter within a column were not significantly different at the 5% level.

### DNA protection capacity

**Figure 3:**
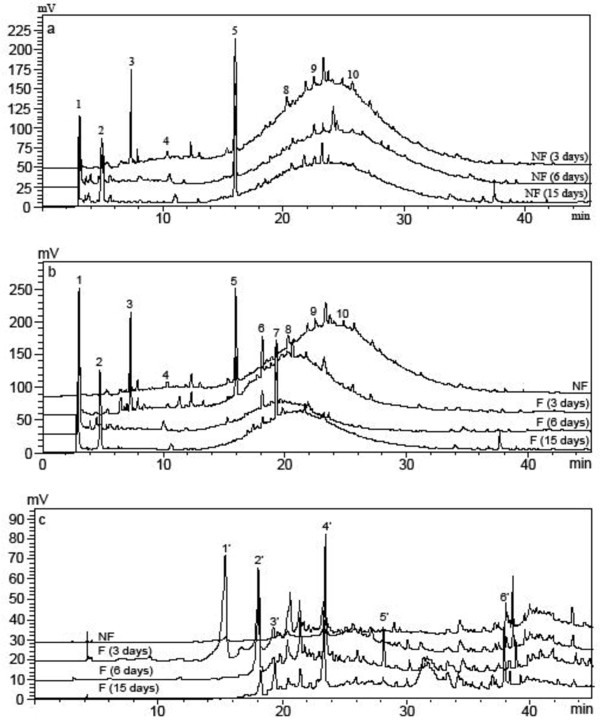
**Electrophoretic patterns of plasmid DNA in the presence of LPE.A**, water; **B**, water and ethanol; **C**, water and damage solution; **D**, ethanol and damage solution; **E**, 100 μg/ml ascorbic acid and damage solution (negative control); **F**, the water-extracted fraction of LPE with damage solution; **H**, **J** and **L**, the water-extracted fractions of the *A. awamori*-fermented LPE (after 3, 6 and 15 days of fermentation) with damage solution; **G**, the ethyl acetate-extracted fraction of LPE with damage solution; and **I**, **K** and **M**, the ethyl acetate-extracted fraction of the *A. awamori*-fermented LPE (3, 6 and 15 days) with damage solution.

Some human diseases such as cancer and neurodegenerative disease involve in imbalance between oxidant and antioxidant defense system. Oxidative DNA damage caused by reactive oxygen species including hydroxyl radical, superoxide anion, and hydrogen peroxide are responsible for these diseases [16]. In this study, DNA protection capacity was used to further investigate the effect of biotransformation on antioxidant capacity of *A. awamori*-fermented LPE. Plasmid DNA has three forms on agarose gel electrophoresis, namely supercoiled circular DNA, open circular form, and linear form. The ·OH radicals were able to cleave DNA strand (Lanes C and D) resulting in the cleavage of supercoiled circular DNA to open circular and linear forms [17]. As shown in Figure 3, the water fraction of the *A. awamori*-fermented LPE showed a better DNA protection ability (Lanes H, J and L), compared with non-*A. awamori*-fermented LPE (Lane F). The DNA protection ability enhanced after 6 days of fermentation (Lane J) and then decreased after 15 days (Lane L). HPLC analysis showed that compound 7 appearing on the 6^th^ day during fermentation may account for the increased DNA protection of the *A. awamori*-fermented LPE. However, no DNA protection ability was observed in the ethyl acetate fraction of both the *A. awamori*-fermented (Lanes I, K and M) and non-*A. awamori*-fermented LPE (Lane G).

### HPLC analysis

The HPLC chromatographic profiles of *A. awamori*-fermented and non-*A. awamori*-fermented LPE are present in Figure 2. Based on the comparison of the retention time of standard compounds, procyanidin B1 (Peak 5), epicatechin (Peak 8) and epicatechin-3-gallate (Peak 10) were identified in the non-*A. awamori*-fermented LPE, while peaks 4, 6, 9 and 6’ present in the *A. awamori*-fermented LPE were recognized as gallic acid, catechin, quercetin-3-glucoside and quercetin, respectively. However, some minor peaks were not identified due to lack of standard compounds in this present study. As shown in Figure 2a, a decrease of compound 3 along with an increase of compound 2 was observed in the non-*A. awamori*-fermented LPE in the fermentation period, possibly due to compound degradation or oxidization. Figure 2b presents the HPLC profile of the water fraction LPE during fermentation by *A. awamori*. The contents of compounds 1 and 2 increased to the maximum value on the 6^th^ days and then decreased slightly. Meanwhile, compound 6 appeared on the 3^rd^ day and then decreased continuously while compound 7 appeared on the 6^th^ day and then increased continuously within 15 days. Figure 2c presents the HPLC profile of the ethyl acetate fraction of LPE during fermentation by *A. awamori*. A decrease of compound 1’ along with an increase of compound 2’ was observed on the 6^th^ day while the maximum contents of compounds 2’, 4’ and 5’ were obtained on the 6^th^ day. Furthermore, the contents of compounds 3’ and 6’ reached the maximum values after 15 days of fermentation. In addition, in the culture extract of the Czapek-Dox medium of *A. awamori* without LPE, only a single peak was observed after 3 min of elution and no other peaks were found in further elution (date not shown), which drew a conclusion that the fungus could not synthesize those phenolic compounds such as compounds 6, 7, 1’, 2’ and 5’ appearing in the *A. awamori*-fermented LPE. Regarding some unknown compounds, further investigation is needed to identify them in the fermented LPE by *A. awamori*.

## Discussion

In this study, *A. awamori* was selected as the starter microorganism to biotransformed phenolic-rich LPE. After fermentation, the phenolic content of the water fraction dramatically decreased within the first 6 days and then increased on the15^th^ days. Degradation and absorption of phenolics available used as a carbon source by *A. awamori* to maintain its growth may account for the decrease in the phenolic content within the first 6 days [18]. Natural degradation of phenolics in LPE without *A. awamori* was observed (data not shown). This is consistent with the finding of Huang et al. [19], who reported decrease of total phenolic content in black soybean koji during storage. Previous reports [12,20,21] pointed out that some hydrolases (e.g. β-glucosidase, β-Xylosidase and α-arabinofuranosidase) present in *A. awamori* were able to crack the linkages between phenolics and their glycosides to produce more hydrophobic compounds, which might account for the increased hydrophobic phenolic compounds in the present study. Similarly, a change in the content of flavonoid content of the water fraction of LPE was observed (Figure 1). Hund et al. [14] and Stoilova et al. [22] reported that some flavanoid-degrading enzymes such as quercetin 2,3-dioxygenase was present in *A. niger* or its variant. These enzymes can cleave C2 and C3 positions in B ring of quercetin to form 2-protocatechuoylphloroglucinol carboxylic acid [23]. The decrease in flavonoid content in the *A. awamori*-fermented LPE might be due to the action of these enzymes.

The effect of *A. awamori* on antioxidant properties of various plant material, such as black bean [24], wheat grain [25] and rice [26], were previously investigated. Among those studies, the increase in phenolic content after fermentation was supposed to be responsible for enhanced antioxidant activity. In this present study, DPPH scavenging activity of the LPE water fraction increased greatly while phenolic content decreased within the first 3 days. Moreover, a slight increase of phenolic content coupled with enhanced DPPH scavenging activity was observed in the LPE ethyl acetate fraction, which indicated that some compounds with higher DPPH scavenging capacity were produced after fermentation.

Plasmid DNA protection of LPE against Feton-reaction mediated breakage was used in this study. The water fraction of *A. awamori*-fermented LPE exhibited much higher DNA protection effect than the non-fermented fraction. However, no such effect was observed from both the *A. awamori*-fermented and non- *A. awamori*-fermented ethyl acetate fractions. Aqueous extract rather than organic solvent extract of plant tissues such as black gram husk [27], *Asplenium ceterach*[28], and areca inflorescence [29] were reported to possess DNA protection activity. In this study, the results concerning DNA protection activity was not completely consistent with DPPH radical scavenging activity. Zhang [30] demonstrated that no significant correlation between DNA protection activity and DPPH radical scavenging activity was observed. Thus, application of DNA protection capacity to antioxidant activity evaluation was needed to be elucidated further.

To further understand the effect of the fermentation on individual phenolic compound, the phenolic profiles of the *A. awamori*-fermented and non-*A. awamori*-fermented LPE were determined by HPLC. The HPLC chromatogram revealed that the main phenolics present in non-*A. awamori*-fermented LPE were procyanidin B1 (Peak 5), epicatechin (Peak 8) and epicatechin-3-gallate (Peak 10), which were also reported by Zhang et al. [31] and Zhao et al. [32]. Great amount of compounds eluted as a hump at the retention time of 15−35 min was in agreement with the report of Roux et al. [33], who demonstrated the hump as proanthocyanidins with different polymerization degree. After the fermentation of *A. awamori*, many peaks disappeared in the water fraction but appeared in the ethyl acetate fraction, which might be attributed to β-glucosidase produced by *A. awamori*[12] as the enzyme can hydrolyse glycosided flavonoids to more hydrophobic aglycones. This assumption was confirmed by the appearance of new compounds (Compounds 1’, 2’ and 4’) after 3 days, and the presence of quercetin (Peak 6’) and the absence of quercetin-3-glucoside (Peak 9) after 6 days of fermentation. In this study, *A. awamori* could cleavage the C4 and C8 bond of procyanidin B1 and then form catechin and epicatechin which reduced the content of compound 5 but increased the contents of compound 6 (catechin) and compound 7 (unknown compound) on the 3^rd^ day of the fermentation (Figure 2b). Meanwhile, B-type procyanidin and catechin were reported to be further metabolized to more hydrophobic A-type procyanidin by some phenol oxidase [34,35], which may account for the appearance of several compounds in the ethyl acetate fraction (Figure 2c).

*A. awamori* generally recognized as safe filamentous fungi [24] was widely present in traditional fermented foods (i.e. miso, natto and tempeh) in East Asia. In the present study, *A. awamori* was used to bioconvert the phenolics-rich LPE. To the best of our knowledge, the study was the first report on the increased antioxidant activity and DNA protection effect in relation to the conversion of phenolics of litchi pericarp by *A. awamori*. This work provided a better way of utilizing litchi pericarp as a readily accessible source of the natural antioxidants in food industry. The fermentation technology can further extend to the bioconversion of phenolic compounds in agriculture-derived by-products. Further investigation into the biochemical pathway of these phenolic compounds converted by *A. awamori* will be needed.

## Materials and methods

### Chemicals

All chemicals used for preparing the Czapek-Dox medium were obtained from Guangzhou Reagent Co. (Guangzhou, China). Gallic acid, catechin, procyanidin B1, epicatechin-3-gallate, quercetin, and quercetin-3-glucoside were purchased from Sigma Chemical Co (St. Louis, MO, USA). Methanol used for HPLC analysis was obtained from CNW Technologies Gmbh (Dusseldorf, Germany).

### Litchi pericarp and starter microorganism

Fresh fruit of litchi (*Litchi chinensis* Sonn.) cv. Huaizhi were harvested from a commercial orchard in Guangzhou, China. The fruit were cleaned carefully with distilled water, and then peeled manually. The pericarp was collected, then dried out door, and finally ground into powder by a grinder (DFT-50, Lingda Mechanics Co., Zhejiang, China).

The microorganism *Aspergillus awamori* GIM 3.4 was obtained from Guangdong Culture Collection Center, Guangzhou, China. The fungus was cultured for 3 days on potato dextrose agar (Guangdong Huankai Microbial Science & Technology Co., Ltd, Guangzhou, China) at 30°C, and then the spores were collected by washing the agar surface with sterile distilled water containing 0.1% Tween 80. The spore suspension was finally adjusted to a concentration of ca. 10^6^/ml with sterile distilled water, which was served as inoculum for the fermentation of litchi pericarp.

### Biotransformation of litchi pericarp extract

Litchi pericarp powder (100 g) was extracted twice with 500 ml of 60% (v/v) ethanol coupled with ultrasonic treatment (40 min at 210 w and 30°C). After filtration through Whatman No. 1 filter paper, litchi pericarp extract (LPE) was concentrated at 50°C using a rotary evaporator under vacuum to remove ethanol, and then adjusted to 500 ml with distilled water. Fresh spore suspension (1 ml) of *A. awamori* was incubated with the sterilized (121°C, 30 min) Czapek-Dox medium (100 ml) containing NaNO_3_ (0.3 g), K_2_HPO_4_ (0.1 g), KCl (0.05 g), MgSO_4_.7H_2_O (0.05 g), FeSO_4_ (0.001 g), sucrose (3 g), LPE (15 ml) and distilled water (85 ml). The inoculated flasks were then cultured at 30°C with shaking at 130 rpm. In addition, incubation of fresh spore suspension (1 ml) with the Czapek-Dox medium (100 ml) without the LPE was also conducted under the same condition. After incubation for 3, 6 or 15 days, the fermented product (100 ml) was filtered through the Whatman No. 1 filter paper and then extracted twice with ethyl acetate (50 ml). The ethyl acetate fraction was concentrated under vacuum and then re-dissolved with absolute ethanol (50 ml), while the hydrophilic fraction was vacuum concentrated to 50 ml. These fractions were collected and then stored at 4°C for further analyses.

### Measurements of total phenolic and flavonoid contents

The measurement of total phenolic content was conducted by the method as described by Quettier-Deleu et al. [36], with some modifications. An aliquot (0.1 ml) of these samples was mixed with 5 ml of distilled water and 0.5 ml of Folin–Ciocalteu phenol reagent. After incubation for 8 min, 20% Na_2_CO_3_ (1.5 ml) were added and heated in boiling water for 1 min. Absorbance was recorded at 760 nm at 25°C in dark. Gallic acid was used to make a calibration curve. Phenolic content was expressed as mg gallic acid equivalent per gram on a dry weigh basis (mg GAE/g DW). All testes were conducted in triplicate.

The total flavonoid content was determined according to the method of Jia et al. [37], with some modifications. Sample or (+)-catechin standard (1 ml) was mixed with 4 ml of distilled water and 0.5 ml of 5% sodium nitrite. After incubation for 6 min, 0.5 ml of 10% aluminum nitrate was added, and then allowed to stand for another 5 min before 4 ml of 1 M NaOH were added. The absorbance was measured immediately against the methanol blank at 510 nm using a spectrophotometer (Uico Shangshai Instrument Co. Ltd., Shanghai, China) in comparison with the standard (+)-catechin. Total flavonoid content was expressed as mg catechin equivalent per gram on a dry weigh basis (mg CE/g DW). All testes were conducted in triplicate.

### Assay of DPPH radical scavenging activity

The method described by Sánchez-Moreno et al. [38] was used to assess the scavenging activity of DPPH free radicals. Briefly, samples were diluted to 100 μg of total phenolics per ml, and then an aliquot (0.1 ml) of the diluted sample was mixed with 2.9 ml of 0.1 mM DPPH solution in methanol. The absorbance was measured at 517 nm after 30 min of incubation at 25°C. The control was carried out with water or ethanol instead of the tested sample, and methanol instead of DPPH was used as blank. The antiradical activity (AA) of the tested sample was calculated using the following formula:

$$\text{AA (\%})=\left(1-\frac{\text{absorbance of sample- absorbance of blank}}{\text{absorbance of control}}\right)\times 100$$

### Protection against DNA breakage

The assay was conducted to determine the protective ability of the *A. awamori*-fermented and non-*A. awamori*-fermented LPE against supercoiled DNA by the method of Kang et al. [17], with some modifications. *Escherichia coli* DH5a cells were transformed with pUC19 plasmid DNA and then grown overnight in the LB medium containing ampicillin (50 μg/ml) at 37°C. Plasmid DNA was purified using the UNIQ-10 Plasmid Kit (Wuhan Sikete Science & Technology Development Co., Ltd., China). The damage solution (1 mM ·OH) was prepared by adding 3.1 μl of 30% H_2_O_2_ into 100 ml of 1 mM FeSO_4._ The reaction solution consisted of 5 μl of plasmid DNA, 5 μL of damage solution, and 5 μl of the tested sample. Three microlitres of loading buffer [30 mM EDTA, 36% (v/v) glycerol, 0.05% (w/v) xylene cyanol FF and 0.05% (w/v) bromophenol blue] were added after 1 hour of incubation in dark, and the reaction products were then electrophoresized in 1% of agarose gel for 40 min under 120 V condition. Agarose gel was stained with 0.05% (w/v) ethidium bromide and then analyzed with image analyzer (Image station 2000R, Kodak, New York, USA).

### HPLC analysis of phenolic compounds

All samples were filtered through a membrane with 0.45-μm pore size prior to injection. Separation, analysis and quantification of phenolic compounds were performed using a Shimadzu LC-20 AT (Shimadzu Corporation, Japan) separation module coupled with a Vydac C18 column (218 TP, 250 × 4.6 mm, 5 μm of particle size, Sigma-Aldrich, St. Louis, MO, USA) and a SPD-10A UV–VIS detector by the method of Yang and Zai [39]. Solvent A (0.1% trifluoroacetic acid) and B (methanol) were used as the mobile phases, with a flow rate of 1 ml/min. The gradient elution program was as follows: 0–5 min, 10% B; 5–35 min, 10–100% B; 35–40 min, 100% B; and 40–45 min, 10% B. The temperature of the column was maintained at 25°C. The injection volume was 20 μl. The chromatogram was recorded at 280 nm. Identification of phenolic compounds was estimated on the basis of retention time.

### Statistical analysis

Data were expressed as means ± standard deviations (SD) and then analyzed by OriginPro 8 (OriginLab Corporation, Massachusetts,USA). The graphs were done using Sigmaplot (Systat Software Inc., San Joes. CA). One-way analysis of variance (ANOVA) with Turkey’s post-hoc test was applied for multiple comparisons. Differences between means at the 5% level were considered significant.

## Conclusions

The results demonstrated that increased antioxidant activity was produced after fermentation of LPE with *A. awamori*. The water-extracted fraction of the fermented LPE possessed more DNA protection capacity. HPLC analysis showed that some new compounds such as catechin and quercetin appeared after of *A. awamori* fermentation of LPE, which could account for the increased antioxidant activity and enhanced DNA protection capacity.

## Competing interests

The authors declare that they have no competing interests.

## Authors’ contributions

SL made a significant contribution to acquisition of data, analysis and manuscript preparation. BY has made a substantial contribution to experimental design and data analysis. FC participated in study design and manuscript revision. GJ participated in partial experiments. QL participated in partial experiments. XD participated in experimental design and data analysis. YJ made a significant contribution to experimental design, data analysis, and manuscript revision. All authors read and approved the final manuscript.
